# Structure Design of Polymer-Based Films for Passive Daytime Radiative Cooling

**DOI:** 10.3390/mi13122137

**Published:** 2022-12-02

**Authors:** Mu Du, Maoquan Huang, Xiyu Yu, Xingjie Ren, Qie Sun

**Affiliations:** 1 Institute for Advanced Technology, Shandong University, Jinan 250061, China; 2 MOE Key Laboratory of Thermo-Fluid Science and Engineering, Xi’an Jiaotong University, Xi’an 710049, China; 3 Institute of Thermal Science and Technology, Shandong University, Jinan 250061, China

**Keywords:** radiative cooling, porous structure, RDSP, passive cooling

## Abstract

Passive daytime radiative cooling (PDRC), a cooling method that needs no additional energy, has become increasingly popular in recent years. The combination of disordered media and polymeric photonics will hopefully lead to the large-scale fabrication of high-performance PDRC devices. This work aims to study two typical PDRC structures, the randomly distributed silica particle (RDSP) structure and the porous structure, and systematically investigates the effects of structural parameters (diameter *D*, volume fraction $f_{v}$, and thickness *t*) on the radiative properties of the common plastic materials. Through the assistance of the metal-reflective layer, the daytime cooling power $P_{\mathrm{net}}$ of the RDSP structures is slightly higher than that of the porous structures. Without the metal-reflective layer, the porous PC films can still achieve good PDRC performance with $P_{\mathrm{net}}$ of 86 W/m${}^{2}$. Furthermore, the effective thermal conductivity of different structures was evaluated. The single-layer porous structure with optimally designed architecture can achieve both good optical and insulating performance, and it is the structure with the most potential in PDRC applications. The results can provide guidelines for designing high-performance radiative cooling films.

## 1. Introduction

The rapid development of global industry, as well as the improvement of the quality of life, leads to strong refrigeration demand [1,2,3]. Traditional cooling technology not only requires energy consumption but also releases greenhouse gases into the atmosphere. The wide use of traditional active cooling devices has resulted in many environmental problems such as the greenhouse effect and global warming [4]. Finding zero-energy consumption, pollution-free, and high-efficiency renewable cooling technology has become a top priority in light of the current energy crisis [5].

Radiative cooling is a passive cooling technology that uses the large temperature difference between the Earth (300 K) and outer space (3 K) to emit infrared radiation without any energy consumption [6,7,8,9]. Nighttime radiative cooling technology has been realized and systematically studied since the 1970s [10,11,12,13,14,15,16]. However, passive daytime radiative cooling (PDRC) is a more challenging problem. Parasitic heat from the sun and the surrounding environment can reduce PDRC performance at sub-ambient conditions (temperatures below ambient) [17,18]. Taking this into consideration, optically selective films for PDRC should have high solar reflectance (${\bar{R}}_{\mathrm{solar}\;}$) and strong thermal emissivity (${\bar{\varepsilon }}_{\mathrm{LWIR}}$) in the “sky transmission window” (8–13 μm) [19,20]. The optically selective radiative cooling film not only increases emission power to the cold universe but also avoids solar radiation absorption, achieving all-day passive cooling performance.

In recent years, due to their excellent infrared emission ability, ease of fabrication, and low cost, polymer-based radiative cooling films have attracted much attention [21,22,23,24]. They possess great potential in the applications of energy-efficient buildings [25,26,27], the passive cooling of solar cells [28], cooling textiles [29], thermoelectric power generation [30], water collection [31], and other fields. Nanotechnology has been conducted to the PDRC applications to improve the cooling performance. By an optimal design of the microstructure, the spectral selectivity of the film can be improved [32]. According to the previous study [33,34,35], radiative cooling materials can be classified into four groups: multilayer structures, metamaterials, randomly distributed particle structures, and porous structures. Among them, multilayer structures and metamaterials suffer from the greatest manufacturing difficulty and are the most costly. The polymer-based porous structure [36,37] and the randomly distributed particle structure [38,39] are the ones that have the best cooling performance and commercial potential [33]. This is because most polymers, such as polycarbonate (PC), polydimethylsiloxane (PDMS), polymethyl methacrylate (PMMA), polyvinylidene difluoride (PVDF), and polyetherimide (PEI), have a high ${\bar{\varepsilon }}_{\mathrm{LWIR}}$, and submicron air pores in polymers have the potential to improve solar reflectance [40]. The morphologies, concentration, size of the particles or pores, and film thickness all affect the cooling performance. To the best of our knowledge, there is no systematic research of the effects of these factors on the cooling performance of polymer-based porous materials and metamaterials.

**Figure 1:**
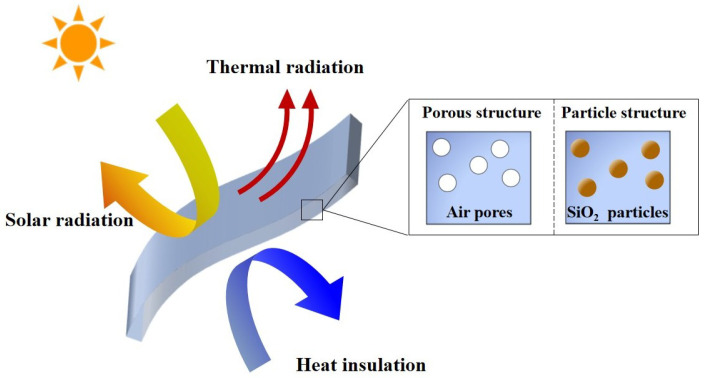
Diagram of radiative cooling film for different structures.

In this study, we used common polymer-based (PC, PDMA, and PMMA) films to design PDRC films with porous structures and randomly distributed particle structures, as shown in Figure 1. From Figure 1, SiO${}_{2}$ was chosen as a typical representative to design randomly distributed particle structures. The effects of the microstructure (diameter *D*, volume fraction $f_{v}$, and thickness *t*) on the apparent radiative properties were investigated through the combination of the Monte Carlo method and the Mie theory. A comparative study of the radiative properties and cooling performance of these two structures was carried out. This work provides guidelines for the application of radiative cooling materials and promotes the large-scale commercialization of radiative cooling films.

## 2. Methods

### 2.1. Radiative Properties of the Films

In this study, PC, PDMS, and PMMA are used as the substrate of the film, and the air micro-pores or silica micro-particles are selected as the dispersion medium. The size parameter χ=πD/λ and the complex refractive index m=n−ik are two important parameters for solving the particle radiative properties. *n* and *k* indicate the refractive index and the absorption index, respectively. When particles are dispersed in the matrix, the normalized refractive index $m_{n}$ of particles in the matrix is calculated by [41]:

(1) $$m_{n}=\frac{n_{m}-ik_{m}}{n_{h}}$$

where the subscript *h* indicates the host medium and subscript *m* indicates the dispersed medium. The extinction efficiency factor $Q_{\mathrm{ext}\;}$, scattering efficiency factor $Q_{\mathrm{sca}\;}$, and scattering phase function Φ(Θ) of the particles can be described by Maxwell’s equations and calculated by Mie theory as follows [42,43]:

(2) Qext(m,χ)=2χ2∑n=1∞(2n+1)Rean+bn

(3) $$Q_{\mathrm{sca}\;}\left(m,\chi \right)=\frac{2}{\chi^{2}}\sum_{n=1}^{\infty }\left(2n+1\right)\left({\left|a_{n}\right|}^{2}+{\left|b_{n}\right|}^{2}\right)$$

(4) $$\Phi \left(\Theta \right)=2\frac{i_{1}+i_{2}}{\chi^{2}Q_{\mathrm{sca}\;}}$$

where $a_{n}$, $b_{n}$ are Mie scattering coefficients calculated by:

(5) $$a_{n}=\frac{\psi_{n}^{\prime }\left(m\chi \right)\psi_{n}\left(\chi \right)-m\psi_{n}\left(m\chi \right)\psi_{n}^{\prime }\left(\chi \right)}{\psi_{n}^{\prime }\left(m\chi \right)\xi_{n}\left(\chi \right)-m\psi_{n}\left(m\chi \right)\xi_{n}^{\prime }\left(\chi \right)}$$

(6) $$b_{n}=\frac{m\psi_{n}^{\prime }\left(m\chi \right)\psi_{n}\left(\chi \right)-\psi_{n}\left(m\chi \right)\psi_{n}^{\prime }\left(\chi \right)}{m\psi_{n}^{\prime }\left(m\chi \right)\xi_{n}\left(\chi \right)-\psi_{n}\left(m\chi \right)\xi_{n}^{\prime }\left(\chi \right)}$$

(7) $$\xi_{n}=\psi_{n}+i\chi_{n}$$

where $\psi_{n}$ and $\chi_{n}$ are the Riccati–Bessel function and satisfy the following relationship.

(8) $$\psi_{n+1}\left(x\right)=\frac{2n+1}{x}\psi_{n}\left(x\right)-\psi_{n-1}\left(x\right),\;\chi_{n+1}\left(x\right)=\frac{2n+1}{x}\chi_{n}\left(x\right)-\chi_{n-1}\left(x\right)$$

(9) $$\psi_{-1}\left(x\right)=\cos x,\psi_{0}\left(x\right)=\sin x,\chi_{-1}\left(x\right)=c-\sin x,\chi_{-1}\left(x\right)=\cos x$$

The extinction coefficient $\beta_{m}$, the scattering coefficient $\sigma_{s,m}$, and the absorption coefficient $\kappa_{m}$ of uniform size particles can be calculated by:

(10) $$\beta_{m}=NC_{e}=NQ_{\mathrm{ext}}G=\frac{6f_{v}}{\pi D^{3}}\times Q_{\mathrm{ext}}\times \frac{\pi D^{2}}{4}=1.5Q_{\mathrm{ext}}\frac{f_{v}}{D}$$

(11) $$\sigma_{s,m}=NC_{s}=NQ_{\mathrm{sca}}G=\frac{6f_{v}}{\pi D^{3}}\times Q_{\mathrm{sca}}\times \frac{\pi D^{2}}{4}=1.5Q_{\mathrm{sca}}\frac{f_{v}}{D}$$

(12) $$\kappa_{m}=\beta_{m}-\sigma_{s,m}$$

The total scattering coefficient $\sigma_{\lambda }$, and the absorption coefficient $\kappa_{\lambda }$ of the film, are expressed as:

(13) $$\sigma_{\lambda }=\sigma_{s,m_{,N}}$$

(14) $$\kappa_{\lambda }=f_{v}\kappa_{m,\lambda }+\left(1-f_{v}\right)\kappa_{h,N}$$

where $\kappa_{h,\lambda }=4\pi k_{h}/\lambda$ is the spectral absorption coefficient of the matrix.

### 2.2. Radiative Transfer Model

The forward one-dimensional Monte Carlo method is applied to solve the radiative transfer equation (RTE) and predict the apparent radiative properties of the films. RTE can be expressed as:

(15) $$\mu \frac{dI_{d}\left(\tau ,\mu \right)}{d_{\mu }}=-I_{d}\left(\tau ,\mu \right)+\frac{\omega }{2}\int_{-1}^{1}P\left(\mu ,\mu^{{}^{\prime }}\right)I_{d}\left(\tau ,\mu^{{}^{\prime }}\right)d\mu^{{}^{\prime }}+\frac{\omega }{4\pi }P\left(\mu ,\mu_{0}\right)F_{0}e^{-\tau }$$

where μ=cos(θ) is the consine of the polar angle θ. We take $\mu_{0}=\cos\left(\theta_{0}\right)=1$ to represent the normal incident. τ=βz is the optical coordinate (β is the extinction coefficient, $\tau_{0}=\beta L$ is the optical depth, and *L* is the thickness of the medium), $I_{d}$(τ,μ) is the diffuse intensity, ω is the single scattering albedo, P(μ, $\mu_{0})$ is the azimuthally averaged scattering phase function, and $F_{0}$ is the incident flux.

During the transmission, the photon bundles are scattered or absorbed by the film. The scattering and absorption effects of the film are determined by the radiative properties calculated through the Mie and effective medium theory (EMT). The direction of scattering photon bundles in a uniform medium can be described through the Henyey–Greenstein phase function [44].

(16) $$\Phi_{\mathrm{HG}}\left(\theta \right)=\frac{1}{4\pi }\frac{1-g^{2}}{{\left(1+g^{2}-2g\cos\theta \right)}^{3/2}}$$

where *g* is the asymmetry factor of the sphere particle described as:

(17) $$g=\langle \cos\theta \rangle =\frac{1}{4\pi }\int_{4\pi }\Phi_{p}\left(r,\theta \right)\cos\theta d\Omega$$

The effective refractive index of films $n_{\mathrm{eff}}$ was calculated by EMT, which is expressed as:

(18) $$\frac{n_{\mathrm{eff}}-n_{m}}{n_{\mathrm{eff}}+2n_{m}}=f_{v}\frac{n_{h}-n_{m}}{n_{h}+2n_{m}}$$

In the present study, the numerical convergence is achieved when the number of photon bundles is larger than 10,000. To make the simulation more accurate, 1 million photon bundles were used for all the MC simulations.

To evaluate the radiative cooling performance of the films, the “sky window” emissivity ${\bar{\varepsilon }}_{\mathrm{LWIR}}$ and the solar reflectance ${\bar{R}}_{\mathrm{solar}\;}$ are calculated. ${\bar{\varepsilon }}_{\mathrm{LWIR}}$ is calculated as the ratio of the cooler emission intensity across the “sky window” (8–13 μm) to the blackbody emission intensity in the same range. ${\bar{R}}_{\mathrm{solar}\;}$ under the AM 1.5 solar spectral irradiance is used to represent the solar reflectance of the film. ${\bar{\varepsilon }}_{\mathrm{LWIR}}$ and ${\bar{R}}_{\mathrm{solar}\;}$ are defined as [45]:

(19) $${\bar{\varepsilon }}_{\mathrm{LWIR}}=\frac{\int_{8\mu m}^{13\mu m}I_{\mathrm{bb}}\left(T,\lambda \right)\varepsilon \left(T,\lambda \right)d\lambda }{\int_{8\mu m}^{13\mu m}I_{\mathrm{bb}}\left(T,\lambda \right)d\lambda }$$

(20) $${\bar{R}}_{\mathrm{solar}\;}=\frac{\int_{0.3\mu m}^{2.5\mu m}I_{\mathrm{AM}1.5}\left(\lambda \right)R\left(\lambda \right)d\lambda }{\int_{0.3\mu m}^{2.5\mu m}I_{\mathrm{AM}1.5}\left(\lambda \right)d\lambda }$$

where $I_{\mathrm{bb}}\left(T,\lambda \right)$ is the spectral intensity emitted by a standard blackbody with a temperature of *T*, $I_{\mathrm{AM}1.5}$ is the AM1.5 spectral solar irradiance, ε(T,λ) is the spectral emittance of film, and R(λ) is the spectral reflectance of film.

### 2.3. Net Radiative Cooling Power

In present study, the film surface temperature *T* and the ambient temperature $T_{\mathrm{amb}}$ are assumed as *T* = $T_{\mathrm{amb}}$ = 300 K. The net radiative cooling power of a film $P_{\mathrm{net}\;}$ can be calculate by [46]:

(21) $$P_{\mathrm{net}\;}\left(T\right)=P_{\mathrm{rad}\;}\left(T\right)-P_{\mathrm{atm}\;}\left(T_{\mathrm{amb}\;}\right)-P_{\mathrm{solar}\;}-P_{\mathrm{non}-\mathrm{radiative}\;}$$

where $P_{\mathrm{net}\;}$ is the net cooling power, $P_{\mathrm{rad}\;}\left(T\right)$ is the radiation power of the surface to the outer space, $P_{\mathrm{atm}\;}\left(T_{\mathrm{amb}}\right)$ is the absorbed power from the atmosphere radiation, $P_{\mathrm{solar}}$ is the absorbed solar radiation energy, and $P_{\mathrm{non}-\mathrm{radiative}\;}$ is the thermal loss power due to the conduction and convection, which can be calculated by the following equations [46]:

(22) $$P_{\mathrm{rad}}\left(T\right)=\int_{2\pi }d\Omega \cos\theta \int_{0}^{\infty }d\lambda I_{\mathrm{bb}}\left(T,\lambda \right)\varepsilon \left(\lambda ,\theta \right)$$

(23) $$P_{\mathrm{atm}}\left(T_{\mathrm{amb}}\right)=\int_{2\pi }d\Omega \cos\theta \int_{0}^{\infty }d\lambda I_{\mathrm{bb}}\left(T_{\mathrm{amb}},\lambda \right)\varepsilon \left(\lambda ,\theta \right)\varepsilon_{\mathrm{atm}}\left(\lambda ,\theta \right)$$

(24) $$P_{\mathrm{solar}}=A\int_{0}^{\infty }d\lambda \alpha \left(\lambda ,0\right)I_{\mathrm{AM}1.5}\left(\lambda \right)$$

(25) $$P_{\mathrm{non}-\mathrm{radiative}\;}=h_{c}\left(T_{\mathrm{amb}}-T\right)\;\;\mathrm{with}\;\;h_{c}=h_{\mathrm{conv}\;}+h_{\mathrm{cond}\;}$$

where $h_{c}$ is a comprehensive non-radiative heat transfer coefficient considering both convection and conduction effect. The effective media theory [47] was employed to describe the effective thermal conductivity of the films:

(26) $$k_{\mathrm{eff}}=k_{h}\frac{k_{m}f_{v}^{\frac{2}{3}}+\left(1-f_{v}^{\frac{2}{3}}\right)k_{h}}{k_{m}\left(f_{v}^{\frac{2}{3}}-f_{v}\right)+\left(1-f_{v}^{\frac{2}{3}}+f_{v}\right)k_{h}}$$

where $k_{h}$ is the thermal conductivity of the host medium and $k_{m}$ is the thermal conductivity of the dispersion medium.

## 3. Results and Discussion

### 3.1. Effect of Diameter on Radiative Properties

**Figure 2:**
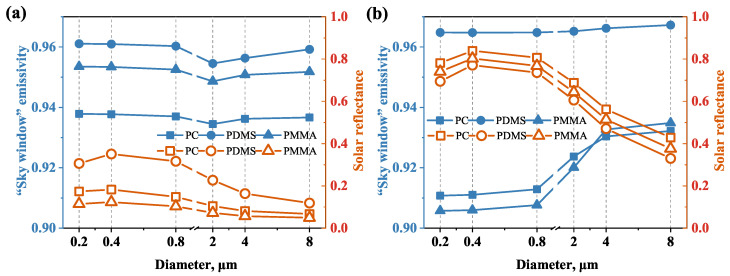
Effect of diameter on the radiative properties of RDSP structure (**a**) and porous structure (**b**).

To carry out an optimal design of the radiative cooling films, the relationship between the apparent radiative properties and the structural parameters needs to be studied first. Selecting a suitable diameter *D* of the dispersion medium can affect the solar reflectance and improve the cooling performance. The volume fraction $f_{v}$ = 0.1 and film thickness *t* = 150 μm are selected to investigate the effect of dispersion medium size on the radiative properties. Figure 2a,b show the radiative properties of the radiative cooling films varying in dispersion medium diameter for different structures (RDSP structure and porous structure). When the diameter *D* increases from 0.2 μm to 8 μm, the solar reflectance tendencies for PC, PDMS, and PMMA films with the RDSP structure or porous structure are consistent. The solar reflectance of the films in both structures reaches its peak value around *D* = 0.4 μm, and we find that higher solar reflectance is achieved by the porous structures, due to the sub-micron air pores, which act as scattering centers of the sunlight and lead to a huge back-scattering effect. The “sky window” emissivity of films with both RDSP and porous structures can be higher than 0.9 due to the high absorbability of the substrate materials in the 8–13 μm range. In addition, we find that the maximum difference in “sky window” emissivity with different diameters does not exceed 0.05 in the range of *D* = 0.2–8 μm for both structures; hence, the diameter has little effect on “sky window” emissivity, and the difference in “sky window” emissivity mainly depends on the material. Therefore, the diameter is set to 0.4 μm to maximize the solar reflectance and maintain a high emittance in the atmospheric window simultaneously.

### 3.2. Effect of Volume Fraction on the Radiative Properties

**Figure 3:**
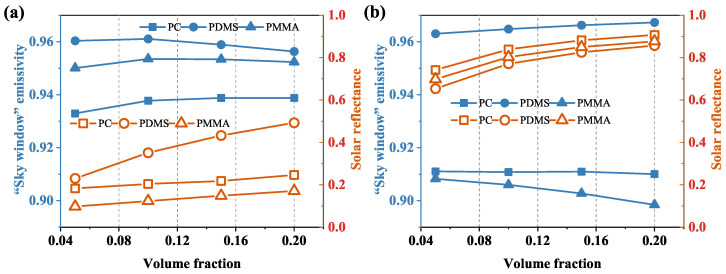
Effect of volume fraction on the radiative properties of different structures. (**a**) RDSP structure and (**b**) porous structure.

The volume fraction $f_{v}$ of the dispersion medium also has an effect on the cooling performance. The diameter of the dispersed medium *D* is set to 0.4 μm, and the thickness of the films *t* is set to 150 μm to investigate the effects of the concentrations of the dispersed medium on the radiative properties. The emissivity and reflectance of films varying with $f_{v}$ for different structures (RDSP structure and porous structure) are shown in Figure 3a,b. The variation trends of the RDSP structure and the porous structure are consistent, increasing with the rise in $f_{v}$. This is because the absorption index of silica in the solar spectrum is quite small and the air sub-micron pores have a strong back-scattering ability, which can improve sunlight reflection. Doping silica particles and air pores in the film increases the scattering centers. As the volume fraction increases, the interface expands, and the mismatch of the refractive index increases the scattering effect. Due to the larger mismatch of the refractive index between the air pores and the polymer, the back-scattering effect is stronger, resulting in a much higher solar reflectance of the porous structure than the RDSP structure. When the $f_{v}$ of air pores is increased to 0.2, the solar reflectance of porous PC, PDMS, and PMMA is close to 0.9. In the volume fraction range of $f_{v}$ = 0.05–0.2, “sky window” emissivity is above 0.89 for both structures and hardly varies with the concentration of the dispersion medium. Unlike PC and PDMS, increasing the concentration of pores weakens the “sky window” emissivity of PMMA, as observed in Figure 3b. This shows that for PMMA porous films, increasing the proportion of dispersion medium may have the opposite effect.

### 3.3. Effect of Thickness on the Radiative Properties

**Figure 4:**
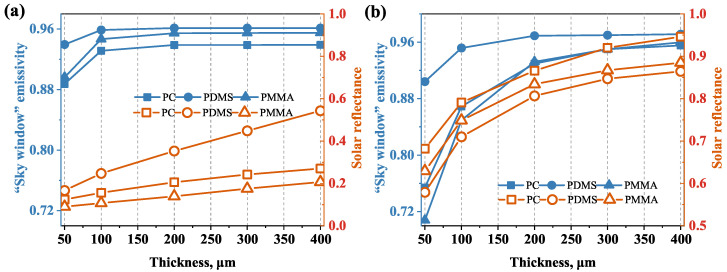
Effect of thickness on the radiative properties of different structures. (**a**) RDSP structure and (**b**) porous structure.

Aside from the size and volume fraction of the dispersion medium, the thickness *t* of the radiative cooling film has a significant impact on the cooling performance. With volume fraction $f_{v}$ = 0.1 and diameter *D* = 0.4 μm, the effect of film thickness is investigated. The radiative properties of the films with different thicknesses for RDSP structure and porous structure are shown in Figure 4a,b, respectively. Since the propagation distance of light increases with the increaseinf thickness *t*, both the solar reflectance and “sky window” emissivity of PC, PDMS, and PMMA films with the RDSP structure or porous structure increased with the increasing film thickness. When the thickness *t* increases from 50 μm to 400 μm, the solar reflectance of the structures improves remarkably. For a porous structure, the solar reflectance of PC, PDMS, and PMMA films reached the maximum when *t* = 400 μm, comprising 0.95, 0.86, and 0.88, respectively. Therefore, increasing the thickness of the structures can effectively improve the PDRC performance, especially the solar reflectivity.

### 3.4. Effect of the Microstructure on Cooling Power

To obtain a good reflectivity of solar radiation, an ideal reflective layer (solar reflectivity of 1) was selected because it exhibits high reflectivity in the solar spectrum. We calculated the net radiative cooling power $P_{\mathrm{net}}$ of the PDRC structures with the reflective layer in Supporting Information. From Figure S3, it is found that the $P_{\mathrm{net}}$ with the RDSP structure is slightly higher than that of the $P_{\mathrm{net}}$ with the porous structure. The maximum values of $P_{\mathrm{net}}$ reached 100 W/m${}^{2}$ and 90 W/m${}^{2}$ for the RDSP structure and porous structure, respectively. This indicates that if the radiative cooling film has relative low solar reflectance, adding a metal-reflective layer is an effective way to improve the daytime radiative cooling performance. However, the high cost and poor durability of the metal-reflective layer also limits its application in radiative cooling.

**Figure 5:**
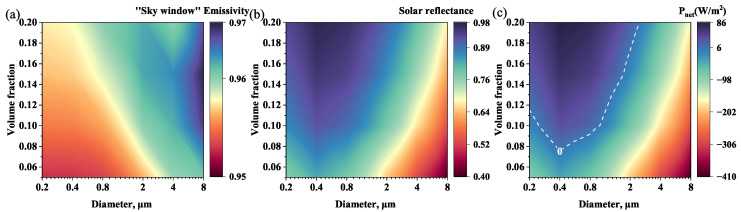
Effect of the microstructure of PC porous films on (**a**) “sky window” emissivity ${\bar{\varepsilon }}_{\mathrm{LWIR}}$, (**b**) solar reflectance ${\bar{R}}_{\mathrm{solar}\;}$, and (**c**) net cooling power $P_{\mathrm{net}}$.

**Figure 6:**
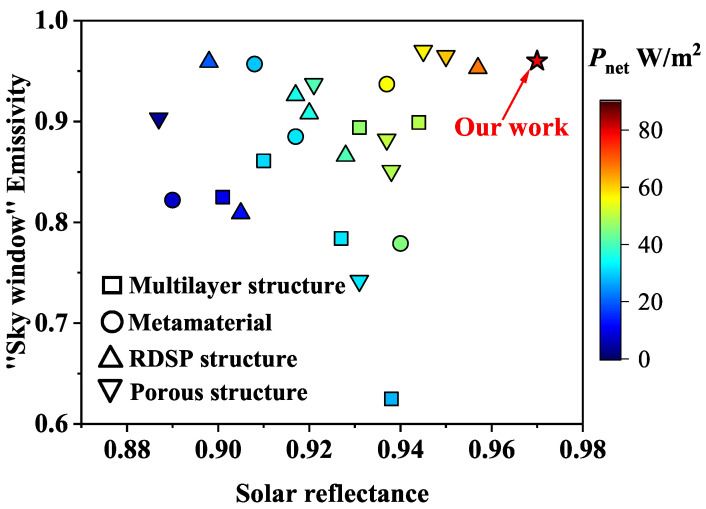
Color map of cooling performance for different PDRC structures.

In general, the sub-ambient daytime radiative cooling can be achieved when ${\bar{R}}_{\mathrm{solar}\;}$> 0.95 (or at least > 0.9) and ${\bar{\varepsilon }}_{\mathrm{LWIR}}$> 0.9 (or at least >0.7) [19,34,48]. The effects of different structural parameters on the ${\bar{\varepsilon }}_{\mathrm{LWIR}}$, ${\bar{R}}_{\mathrm{solar}\;}$, and $P_{\mathrm{net}}$ were calculated for a porous PC porous film with a thickness of 400 μm. As shown in Figure 5a, ${\bar{\varepsilon }}_{\mathrm{LWIR}}$ varies little with particle size *D* and volume fraction $f_{v}$, remaining between 95% and 97%. In Figure 5b, the maximum value of ${\bar{R}}_{\mathrm{solar}\;}$ appears at the particle size of 0.4 μm and ${\bar{R}}_{\mathrm{solar}\;}$ increases with the increasing $f_{v}$. Since ${\bar{\varepsilon }}_{\mathrm{LWIR}}$ of PC porous film is greater than 0.9 and varies little with $f_{v}$ and *D*, there is not much room to enhance radiation power of the surface ($P_{\mathrm{rad}}$) to improve the $P_{\mathrm{net}}$. The absorbed solar radiation energy ($P_{\mathrm{solar}}$) plays a leading role in $P_{\mathrm{net}}$. Therefore, small-size PC at high $f_{v}$ has a higher ${\bar{R}}_{\mathrm{solar}\;}$, which is easier to achieve high cooling performance. According to the simulation results, the ${\bar{R}}_{\mathrm{solar}\;}$ and ${\bar{\varepsilon }}_{\mathrm{LWIR}}$ of PC porous films (*D* = 0.4 μm, $f_{v}$ = 0.2, and *t* = 400 μm) are 97 and 96, respectively. When $T=T_{\mathrm{amb}}=300$ K, $P_{\mathrm{rad}}$, $P_{\mathrm{atm}}$, and $P_{\mathrm{sun}}$ are 324, 215, and 23 W/m${}^{2}$, respectively. Thus the net cooling power $P_{\mathrm{net}}$ is 86 W/m${}^{2}$. Note that no substrate is used here, and $P_{\mathrm{net}}$ at this time is a conservative estimate. A comparison of the cooling performance of the single-layer porous PC film with several other typical PDRC structures is shown in Figure 6. The cooling power of the different structures is taken from the literature [33]. As shown in Figure 6, the well designed porous structure can achieve high cooling performance without the metal reflecting layer. The single-layer porous structures with high-performance and low-cost have more potential in PDRC application.

### 3.5. Influences of Different Structures on Thermal Insulation Performance

**Figure 7:**
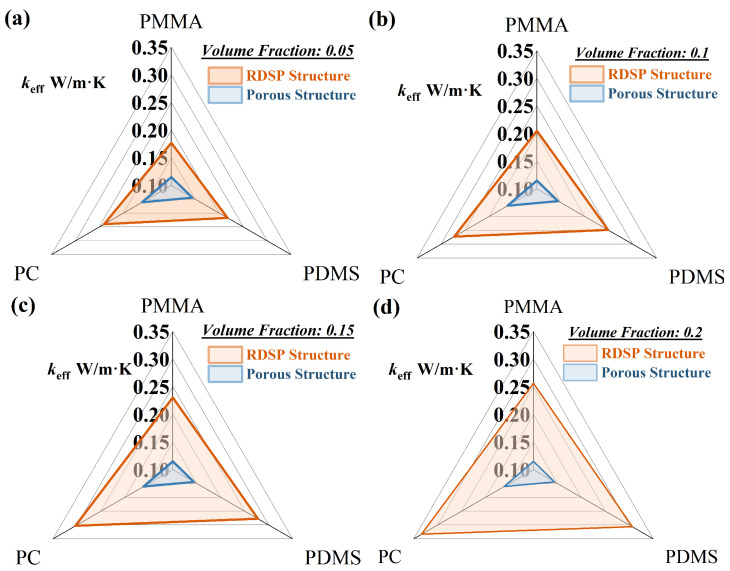
Thermal conductivity of radiative cooling film for different structures: (**a**) $f_{v}$ = 0.05, (**b**) $f_{v}$ = 0.1, (**c**) $f_{v}$ = 0.15, and (**d**) $f_{v}$ = 0.2.

When there is a large temperature difference between the cooling surface and the surrounding environment (300 K), the parasitic heat gain from the surrounding environment will limit the cooling performance of the film. Because air has a lower thermal conductivity (0.026 W/m·K) than silica particles (1.4 W/m·K), air pores can efficiently isolate parasitic heat gain. This section provides a brief discussion of the thermal insulation properties of different structures when the cooling surface temperature is lower than the ambient temperature. The effective thermal conductivity of PC, PDMS, and PMMA films with RDSP and porous structures was calculated using Equation (26). The film with porous structures achieves superior insulating performance than the film with RDSP structures, as observed in Figure 7. As the volume fraction of dispersed medium increases, the thermal conductivity of the porous structure decreases, while the thermal conductivity of the PDRC structure increases. When $f_{v}$ = 0.2, the effective thermal conductivity of the porous PC, PDMA, and PMMA structures is 52%, 53%, and 55% lower than that of the RDSP structure, respectively. This demonstrates that, in the case of a considerable temperature difference between indoor and outdoor environments, the film with a polymer porous structure can effectively block heat from the external environment and maintain the cooling performance of films.

## 4. Conclusions

In present study, we numerically investigated the radiative properties of the radiative cooling films with porous and randomly distributed particle structures. The effects of polymer material, particle/pore size, volume fraction, and film thickness on the radiation properties and cooling performance were studied systematically. The ${\bar{R}}_{\mathrm{solar}\;}$ increases as the volume fraction and thickness increase, peaking at *D* = 0.4 μm. With a fixed disperser size and concentration, the ${\bar{\varepsilon }}_{\mathrm{LWIR}}$ of various structures all increase with increasing thickness, and they tend to be steady at a thickness of 400 μm. After adding the metal substrate, the $P_{\mathrm{net}}$ of the RDSP structure is slightly higher than that of the porous structure, and the $P_{\mathrm{net}}$ of the PC material is slightly higher than that of the PDMS and PMMA materials. The $P_{\mathrm{net}}$ of the porous PC film (*D* = 0.4 μm, $f_{v}$ = 0.2, and *t* = 400 μm) can still reach 86 W/m${}^{2}$ without the metal-reflective layer. The results of this study provide references for developing high-performance radiative cooling films.

## Data Availability

Not applicable.

## Supplementary Materials

The following supporting information can be downloaded at: https://www.mdpi.com/article/10.3390/mi13122137/s1, Figure S1 Radiative properties of radiative cooling films with randomly distributed particles. Figure S2 Comparison of emissivity between the present model and the experimental data [3]. Figure S3 Effect of the microstructure on the daytime cooling power of various materials. (a)–(d): Cooling power of RDSP structures. (e)–(g): Cooling power of porous structures. References [2,49,50] are cited in the supplementary materials.

## Nomenclature

The following nomenclature are used in this manuscript:

$a_{n}$	Mie scattering coefficient
$b_{n}$	Mie scattering coefficient
*D*	diameter of dispersed medium, μm
$f_{v}$	particle volume fraction
*g*	asymmetry factor
$h_{c}$	comprehensive non-radiative heat transfer coefficient
$I_{\mathrm{AM}1.5}$	AM1.5 spectral solar irradiance
$I_{\mathrm{bb}}\left(T,\lambda \right)$	spectral intensity emitted by blackbody
$k_{\mathrm{eff}}$	effective thermal conductivity, W/m·K
*m*	complex refractive index, m=n−ik
$m_{n}$	normalized complex refractive index
$P_{\mathrm{atm}}$	ambient radiation power, W/m${}^{2}$
$P_{\mathrm{net}}$	daytime net cooling power, W/m${}^{2}$
$P_{\mathrm{non}-\mathrm{radiative}}$	thermal loss power, W/m${}^{2}$
$P_{\mathrm{rad}}$	the radiation power, W/m${}^{2}$
$P_{\mathrm{solar}}$	absorbed solar radiation energy, W/m${}^{2}$
$Q_{\mathrm{ext}\;}$	extinction efficiency factor
$Q_{\mathrm{sca}\;}$	scattering efficiency factor
R(λ)	spectral reflectance of film
${\bar{R}}_{\mathrm{solar}\;}$	solar weighted reflectance
*T*	temperature, K
*t*	thickness of films, μm
**Greek symbols**
ε(T,λ)	spectral emittance of film
$\beta_{m}$	extinction coefficient
${\bar{\varepsilon }}_{\mathrm{LWIR}}$	weighted emissivity
$\kappa_{\lambda }$	total absorption coefficient
$\kappa_{m}$	absorption coefficient
λ	wavelength of radiation, μm
$\sigma_{s,m}$	scattering coefficient
$\sigma_{\lambda }$	total scattering coefficient
**Superscripts and subscripts**
amb	ambient
h	host medium
m	dispersed medium
