# Supplementary material for: Structure Design of Polymer-Based Films for Passive Daytime Radiative Cooling
Source: Micromachines (Basel). 2022 Dec 2;13(12):2137. doi: 10.3390/mi13122137 (PMC9782091; doi:10.3390/mi13122137)
Supplement: Supplementary file 1 [file micromachines-13-02137-s001.zip › micromachines-2028486-supplementary.pdf]

Supporting Information

Supporting Information S1: Radiative properties of randomly distributed different particles

Using PC polymer as the substrate, films of randomly doped TiO_2, Al_2O_3, and SiO_2 particles for radiative cooling were designed. Then, the solar reflectance and ”sky window” emissivity were calculated. The diameter of the particles is 0.4 μm, the volume fraction is 0.1, and the thickness of the film is 150 μm. As shown in Fig.S1, the solar reflectance of TiO_2 is much higher than that of Al_2O_3 and SiO_2, but its ”sky window” emissivity is lower compared to the other two materials. In addition, although TiO_2 has a high solar reflectance, it still can not obtain a positive cooling power. We calculated the cooling power of the radiative cooling film with randomly distributed TiO_2, Al_2O_3, and SiO_2 particles with an ideal metal-reflective layer. Their cooling powers were 58, 66, and 83 W/m^2 respectively. Therefore, in this study, SiO_2 was chosen as a typical representative to design randomly distributed particle structures for a systematic comparative study with porous structures.

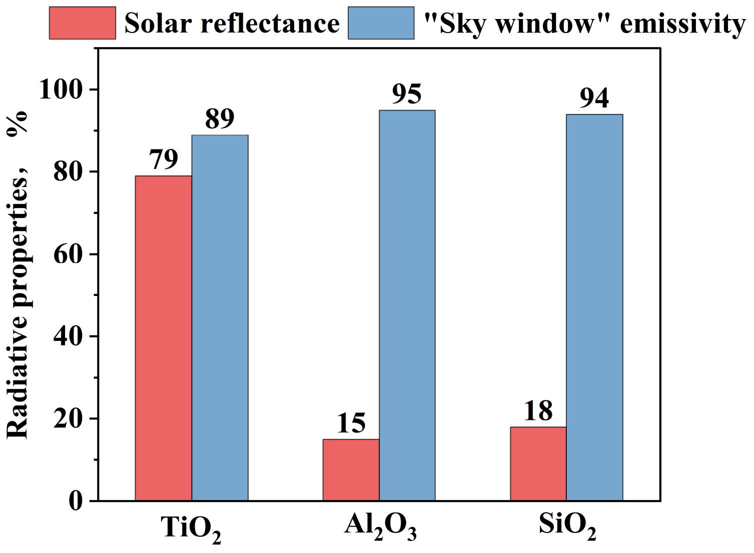

Figure S1. Radiative properties of radiative cooling films with randomly distributed particles.

Supporting Information S2: Model validation

In our previous study [1, 2], the optical results of porous structure were verified with the experimental results. To validate the RDSP structure, we compared the numerical results to experimental data from a PMMA film doped with silica particles published in the literature [3] with the same structural parameters. The diameter of the silica particles is 4 μm, the volume fraction is 6 %, and the film thickness is 70 μm. The emissivity of the ”sky window” $\bar{\varepsilon}_{LWIR}$ of the numerical structures with the same structural parameters was calculated. The simulation results and

experimental data are shown in Fig. S1. The ”sky window” emissivity of the experimental data is 94.3% and the numerical results is 91.7%. The average deviation between the numerical results and the experimental data is less than 3 %, which validates the present theoretical model.

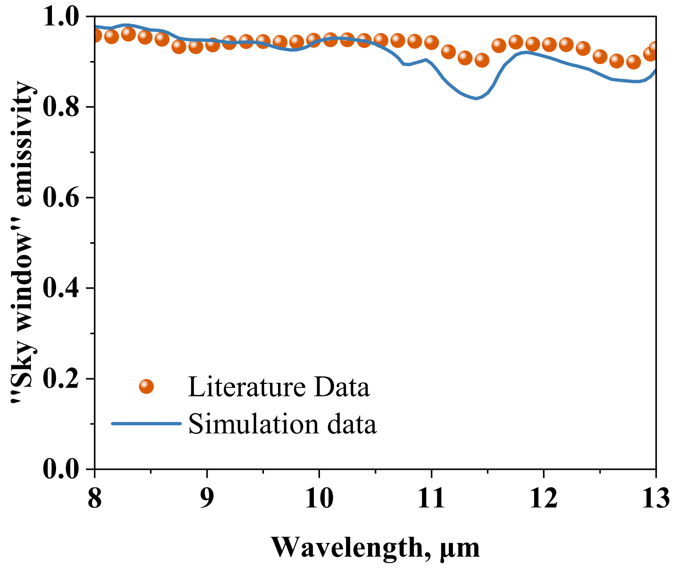

Figure S2. Comparison of emissivity between the present model and the experimental data [3].

Supporting Information S3: Cooling power of radiative cooler with a silver-reflective layer

Figure S3(a)-(d) shows the net cooling power of the film with randomly distributed silica particles. When the diameter and thickness are constants, the P_{net} of the film increased with the increase of the concentration of the doping particles. With a thinner film thickness, this trend will become more obvious. When the concentration of silica particles is constant, the P_{net} of the film with a thickness of 50 μm achieves maximum cooling power at a diameter of 4 μm. And the P_{net} of the film with a thickness of 100 μm or 150 μm increases with the increase of the particle size. Figure S3(e)-(h) show the net cooling power P_{net} of the porous films with different diameters, concentrations and thicknesses. When the disperser diameter and film thickness are fixed, the P_{net} of the film increased with the increase of the doping concentration. With a fixed volume fraction, the P_{net} of the film achieves a maximum value at D = 4 μm. According to the data from Figure S3, it is found that the P_{net} with the RDSP structure is slightly higher than that of the P_{net} with the porous structure. When the thickness is constant, the P_{net} of the micron range(D = 2-8 μm) outperform the P_{net} of the sub-mircon range(D = 0.2-0.8 μm) for both RDSP and porous structures. The P_{net} and f_v of the film are positively correlated in the range of f_v = 0.05-0.2.

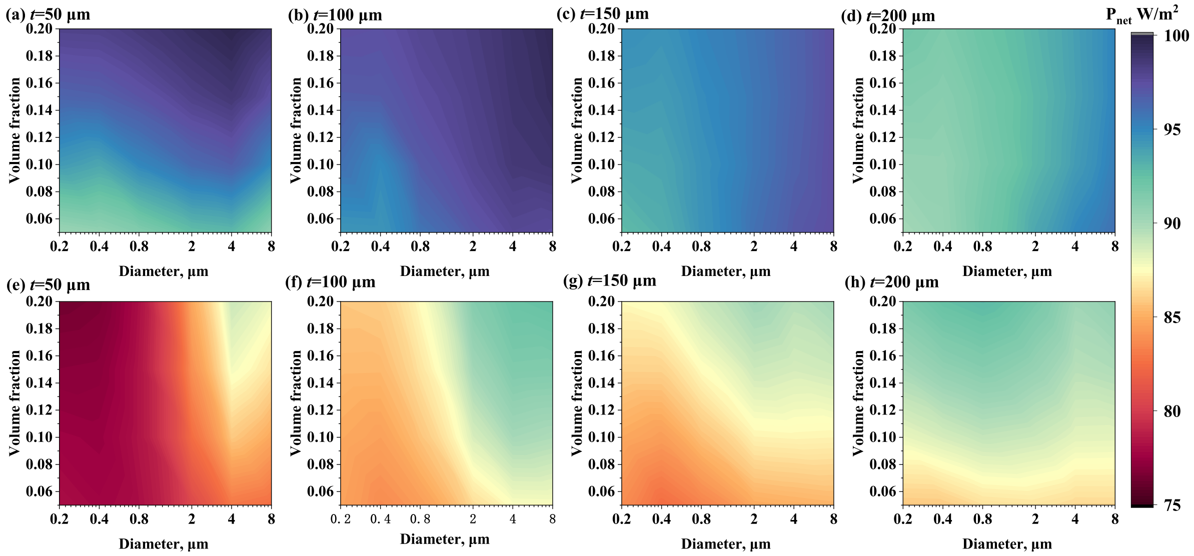

Figure S3. Effect of the microstructure on the daytime cooling power of various materials. (a)-(d): Cooling power of RDSP structures. (e)-(g): Cooling power of porous structures.

References

[1] M.Q. Huang, X.Y. Yu, J.C. Wan, M. Du, X.Y. Wang, Q. Sun, and G.H. Tang. All-day effective radiative cooling by optically selective and thermally insulating mesoporous materials. *Sol. Energy*, 235:170–179, 2022.

[2] X.Y.Yu, M.Q. Huang, X.Y. Wang, G.H.Tang, and M. Du. Plasmon silica aerogel for improving high-temperature solar thermal conversion. *Appl. Therm. Eng.*, 219:119419, 2023.

[3] Z.M. Cheng, F.Q. Wang, Gong D.Y, H.X. Liang, and Y. Shuai. Low-cost radiative cooling blade coating with ultrahigh visible light transmittance and emission within an “atmospheric window”. *Sol. Energy Mater. Sol. Cells*, 213:110563, 2020.
